# Qualification for upper secondary education in individuals with autism without intellectual disability: Total population study, Stockholm, Sweden

**DOI:** 10.1177/1362361320975929

**Published:** 2020-11-27

**Authors:** Isidora Stark, Peiwen Liao, Cecilia Magnusson, Michael Lundberg¹, Dheeraj Rai, Anton Lager, Selma Idring Nordström

**Affiliations:** 1 Karolinska Institutet, Sweden; 2 University of New South Wales (UNSW), Australia; 3 Stockholm County Council, Sweden; 4 University of Bristol, UK

**Keywords:** Attention-deficit hyperactivity disorder, autism, predictors, school outcome, total population cohort

## Abstract

This study used the Stockholm Youth Cohort, a total population cohort (*N* = 364,957), to describe patterns and predictors of qualification for upper secondary education, defined by passing graduation grades in core compulsory school subjects in contemporary young individuals diagnosed with autism spectrum disorders without intellectual disability (*n* = 6138). At the expected age for graduation, 16 years, 29% (adjusted rate difference 95% confidence interval (28.0–30.0)) fewer autistic than non-autistic individuals were qualified for upper secondary education (57% and 86%, respectively). Comorbid attention-deficit hyperactivity disorder further increased this difference. Within the group of autistic students without intellectual disability, female sex and lower family income were associated with non-qualification for upper secondary education. The proportion of students with autism without intellectual disability who qualified for upper secondary education increased at age 20. These findings underline the need for improved support for students with a diagnosis of autism without intellectual disability in mainstream education.

**Lay abstract:**

Obtaining a quality education is important for any individual’s chances of leading a healthy and thriving life. Currently, educational policies in many countries underscore the rights of students with autism to be educated in mainstream schools. While there is some knowledge on school outcomes among students with autism from older studies, little is known about rates of qualification for upper secondary education among children with autism in mainstream schools today. This lack of knowledge is problematic since autism is diagnosed more widely, and prior evidence may not be relevant for individuals with autism and their families today. Using Swedish registers, we therefore examined this in a study including all children and young people in Stockholm County in 2001 through 2011. We found that about two thirds of children with autism without intellectual disability qualified for upper secondary education at the expected age, in comparison with about nine in ten among typically developing peers. We also found that girls with autism had further difficulties obtaining such qualification than boys and that those who were additionally diagnosed with attention-deficit hyperactivity disorder were particularly at risk of non-qualification. Finally, students with autism without intellectual disability had a greater chance of completing compulsory education if given an extended period to graduate. These findings underline the need for supportive interventions for children with autism during compulsory school. They may also challenge the inclusive education policy adopted by majority of western countries, at least in the wake of addressing special needs in mainstream schooling.

The social and academic challenges of school impose particular demands on students with autism spectrum disorders (henceforth autism), a heterogeneous group of neurodevelopmental conditions characterized by difficulties with social interaction and restricted/repetitive patterns of behaviours and interests (American Psychiatric Association, 2013). Recent studies demonstrated a rapid increase in diagnoses of autism without intellectual disability (ID) in Sweden (Idring et al., 2015) and other developed countries (Elsabbagh et al., 2012). It is estimated that almost 2.5% of young people in Stockholm County have a diagnosis of autism, 75% of whom have no ID (Idring et al., 2015). Studies exploring scholastic performance, particularly those examining objectively measured educational outcomes, in this large group of youth are scarce (Keen et al., 2016).

The relationship between educational attainment and health outcomes is complex, as the direction of the observed associations is uncertain and may be confounded by unobserved factors (Gustafsson et al., 2010). Nevertheless, a recent review and meta-analysis of 89 studies concluded that educational attainment has mixed but largely beneficial effects on the number of health outcomes, including mental health (Hamad et al., 2018). Educational attainment is furthermore related to future educational and occupational success (Lê-Scherban et al., 2014).

Studies of educational attainment among students with autism are scarce (Keen et al., 2016), although the interest for better understanding of the outcomes in this population is increasing. Research to date has focused on individual-level skills such as reading comprehension and problem solving (Keen et al., 2016; May et al., 2013). However, knowledge about how these students perform in relation to school-administered tests and official measures including graduation grades which are needed to successfully continue an education is missing (Keen et al., 2016). Furthermore, small sample sizes, non-population-based design and/or short follow-up limit the generalizability of existing findings. Moreover, overall, studies of educational outcomes prior to 1990s suggested a poor prognosis, and approximately 50%–60% of autistic students did not manage to achieve formal academic or vocational qualifications (Levy & Perry, 2011). In a study on outcomes among young adults with autism born between 1974 and 1984 and attending mainstream schooling, only a third attended postsecondary school (Eaves & Ho, 2008). Yet, to inform effective educational interventions for this group today, we need to better understand predictors of educational attainment in autism without ID from more recent birth cohorts.

Predictors of educational attainment such as sex and parental sociodemographic background have been found to have a substantial impact on students’ outcomes in the general population (Sirin, 2005) and might moderate outcomes within the autistic group as well. Annual household income and parental education predicted future educational outcomes such as participation in postsecondary education and employment in high school leavers with autism (Chiang et al., 2012, 2013). However, whether parental characteristics modify school performance in autistic students during other educational stages is unknown.

The effect of sex on educational attainment in individuals with autism remains unclear, and the existing evidence has pointed to different directions of the potential effect. Similar to the sex differences in the general population (Serbin et al., 1990), there is some evidence that girls with autism had superior executive functioning and less stereotypic behaviours compared to males (Bölte et al., 2011). This advantage may lead to better school performance in autistic females. However, there is also evidence that autistic females are more likely to have lower cognitive abilities than males (Frazier et al., 2014), which may imply adverse effects on educational attainment among autistic females.

A considerable proportion of individuals with autism also have co-occurring attention-deficit hyperactivity disorder (ADHD), with difficulties regulating attention, activity and impulsivity (Hofvander et al., 2009; Skokauskas & Gallagher, 2012). ADHD is associated with poor school performance in relation to cognitive capacity (Loe & Feldman, 2007), and individuals with both autism and ADHD may experience additional challenges. Furthermore, there is evidence that treatment of ADHD could be an important focus of intervention aiming to prevent school failure (Baweja et al., 2015). Thus, autistic individuals with comorbid ADHD may be a clinical phenotype within the autistic population that requires particular attention in planning of educational services.

In summary, while educational attainment is an important predictor of life chances including future health, there is a lack of prospective, large population-based studies examining objectively measured outcomes in students with autism without ID from more recent birth cohorts. Here, we utilize prospectively collected data from registers, to investigate the association between autism without ID and qualification for upper secondary mainstream education, measured by required graduation grades. In this recent total population study, we also explore if this association varies with sex and parental sociodemographic characteristics, for example, whether these factors alleviate or aggravate risks for non-qualification in autism without ID. In addition, we study this risk according to the presence of comorbid ADHD in autism without ID, including for how graduation grades in core subjects vary between autistic and non-autistic individuals. Finally, we investigate whether, in Sweden, entitled, prolonged attendance of compulsory school improve chances of graduation in autism without ID by examining qualification for upper secondary education at both the expected (16 years) and the maximum allowed age (20 years) for graduation.

## Methods

### Participants

We conducted an observational, longitudinal, register-based study in the Stockholm Youth Cohort (SYC), a total population cohort of children aged 0–17 years resident in Stockholm County, Sweden, from 2001 to 2011 (*N* = 736,180). A range of national and regional records were linked using personal identification numbers, assigned to every Swedish resident (Ludvigsson et al., 2016). A range of published studies used data from the SYC, and detailed description of the SYC is available elsewhere (Idring et al., 2012).

**Figure 1.**
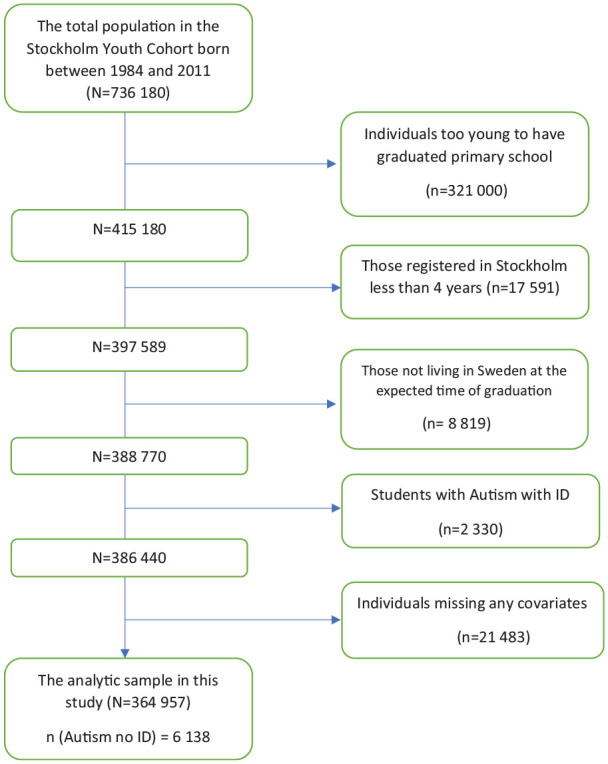
Derivation of analytical sample.

Figure 1 shows the derivation of the analytical sample. Because the expected age of graduation from compulsory school is 16 years, only children born from 1984 through 2000 were included in the study (*N* = 415,180). Individuals attending non-regular compulsory schools (e.g. international schools or schools based on special educational approaches, such as Waldorf schools) and special needs schools (e.g. for students with ID) were not listed in the Register of Compulsory School Leaving Grades and were thereby not included in the study. In 2011/2012, around 15% of all compulsory school students attended non-regular schools (Skolverket, n.d.). To control for educational background, such as differences in educational quality or systems across regions or countries, we excluded individuals who had lived in Stockholm for less than 4 years (*n* = 26,410) and those who did not live in Sweden at the expected time of their compulsory school graduation (*n* = 8819). In our dataset, around 5% of individuals in both the autistic and non-autistic groups were found to have at least one missing covariate (347 and 21,136 individuals, respectively). After excluding those with missing data on any covariates (*n* = 21,483), our final analytic sample consisted of 364,957 individuals, of whom 6138 were diagnosed with autism without ID through 2011.

### Ascertainment of autism in the SYC

Sweden has a publicly funded system for the screening, diagnosis and habilitation of individuals with neurodevel-opmental disorders including autism, ID and ADHD. Neurodevelopmental assessments are typically conducted by child psychiatric or paediatric teams and include evaluations of cognitive-, socio-communicative and adaptive skills (Idring et al., 2012). Diagnoses of autism were ascertained from several registers covering all services providing diagnostic evaluations and care for autism in Stockholm, using International Classification of Diseases (ICD) and *Diagnostic and Statistical Manual of Mental Disorders* (4th ed.; DSM-IV) codes (ICD-9-299, ICD-10 F84 and DSM-IV 299) and by the denotation of autism by habilitation services (Axén et al., 2010). The multisource methodology used to ascertain cases with autism overall was found to be valid through a review of medical records and cross-validation against a twin study, while validity of autism cases by ID status was confirmed through review of medical records (Idring et al., 2012). Diagnoses of ID and ADHD were identified in the same manner (defined as ICD-10-F70–F79, DSM-IV 317–319 for ID; and ICD-9-F90.0, F98.8 and DSM-IV 314.00-314.01 for ADHD). Because autisms are considered as lifelong conditions, we classified individuals as cases of autism without ID when registered with a diagnosis at any time before 31 December 2011. For some analysis, individuals with autism without ID were further categorized according to the presence or absence of comorbid ADHD.

### The Swedish school system and qualification for upper secondary school

Compulsory schooling in Sweden entails 9 years and a separate preschool year, with expected age of 16 at the time of graduation from the final, 9th year. Compulsory schooling is regulated by the Swedish Education Act (SFS 2010:800) and is called compulsory school attendance. Compulsory school attendance ends latest at the age of 18, but the right to complete compulsory schooling is extended two additional years if the student has not attained knowledge goals until the maximum of age 20. While students with ID are entitled to special education in compulsory school – regardless of comorbid conditions (SFS 2010:800) – those with autism without ID and/or ADHD commonly attend education in mainstream compulsory schools. The Swedish Education Act does not mandate support for specific neuropsychiatric diagnoses, but instead promotes adaptation or special support to all students who have difficulties in reaching knowledge requirements (SFS 2010:800).

Students who graduate from the final year of compulsory school may qualify to access upper secondary education. Eligibility to access upper secondary education is based on grades in the final year of compulsory school (henceforth graduation grades). The students’ performance is evaluated throughout schooling by subject grades in 16 subjects included in the Swedish compulsory school system. While entrance requirements vary among upper secondary education programmes, passing grades in three core subjects from the final year of compulsory school is mandatory for all (Swedish Institute, 2019). The grading system is supervised by the National School Administration through national examinations for all students in mainstream schools (Skolverket, 2005). Data on graduation grades are routinely collected and were extracted from the Register of Compulsory School Leaving Grades for this study. Qualification for upper secondary education was our main outcome, defined as having at least a passing grade in three core subjects (Mathematics, Swedish and English) as recorded in the Register for compulsory school leaving grades. We also examined subject-specific grades in the three core subjects as secondary outcomes.

### Covariates

Sociodemographic characteristics identified in previous studies as associated with autism and scholastic performance were collected from various registers as previously described (Idring et al., 2012). Characteristics in our analyses included parents’ ages at child’s birth (Idring et al., 2014), immigration status (born in Sweden with at least one Swedish-born parent, born in Sweden with both parents born abroad, and born outside Sweden; Bankston & Zhou, 2002), familial income (grouped into quintiles after deducting taxes and adjusting for family size and inflation, with 1 being the *lowest* quintile and 5 the *highest*) and parents’ educational level at child’s birth (Sirin, 2005). The highest educational level for either parent was grouped as *primary* (9 years of schooling or less), *upper secondary* (10–12 years) and higher (>12 years). Given the evidence that parental psychiatric conditions may influence both autism (Daniels et al., 2008) and school performance in their children (Ranning et al., 2018), history of any maternal and paternal psychiatric care was retrieved and analysed as a dichotomous variable.

### Statistical analyses

First, descriptive analyses of individual and parental sociodemographic characteristics and selected school outcomes were performed. These characteristics were presented as proportions using chi-square test for categorical variables and independent *t*-test for metric variables to compare differences between the autistic individuals and their non-autistic peers, and within the autistic group for those with and without comorbid ADHD. A *p*-value of 0.05 was set as level of significance for all statistical analyses.

Poisson models with identity-link and robust variance estimates (SAS version 9.4) were used to estimate rate differences (RDs) and corresponding 95% confidence intervals (CIs) between autistic (autism without ID overall, and with or without comorbid ADHD) and non-autistic individuals’ qualification for upper secondary education at age 16 and age 20. Crude analyses were adjusted by sex and birth year. Besides sex and birth year, our full model included immigration status, family disposable income, parental ages and education at birth of the index person and parental psychiatric history. We also estimated RDs between autistic and non-autistic individuals in stratified analyses according to sex, immigration status and family disposable income to explore whether these factors alleviate or aggravate risks for non-qualification in autism without ID. Finally, we examined RDs for subject-specific grades in core subjects in relation to autism without ID and comorbid ADHD.

## Results

### Descriptive statistics

We identified 364,957 individuals old enough to graduate compulsory school from 2001 through 2011, of whom nearly 2% (*n* = 6138) were identified with autism without ID. Among those, 2598 (42%) had comorbid ADHD.

Table 1 shows sociodemographic characteristics of the study population and qualifications for upper secondary education at ages 16 and 20. Those with autism without ID were more likely than non-autistic individuals to be male, born in Sweden, and have at least one Swedish-born parent, to live in families of middle socioeconomic status, and to be born after 1995. Autistic individuals without ID were more likely to have parents who had contact with psychiatric services compared to their non-autistic peers. Autistic individuals with comorbid ADHD were more likely to be male than those without ADHD and less likely to have parents with postsecondary education or to be from families in the highest income quintile. A larger proportion of those identified with both autism without ID and ADHD than those with autism without ID only had parents with a registered psychiatric history. At ages 16 and 20, a significantly lower proportion of autistic individuals (57% and 66%; *p* < 0.05) met eligibility criteria for upper secondary education as compared to their non-autistic peers (86% and 89%; *p* < 0.05). A significantly lower proportion of autistic students with comorbid ADHD met eligibility criteria for upper secondary education at ages 16 and 20, as compared to those without ADHD (52% and 61% vs 62% and 69%, respectively; *p* < 0.05).

**Table 1.** Demographic characteristics of the study population, by autism without intellectual disability (Autism-ID) overall and Autism-ID with and without ADHD (%) and qualification for upper secondary school at ages 16 and 20.

Total = 364,957	Non-autism (*N* = 358,819) %	All Autism-ID (*N* = 6138) %	P1	Autism-ID without ADHD (*N* = 3540) %	Autism-ID with ADHD (*N* = 2598) %	P2
Individual and familial characteristics						
Male	50.88	67.74	<0.001	65.00	71.48	<0.001
Birth year						
1984–1989	32.62	21.00	<0.001	24.52	16.20	<0.001
1990–1994	33.41	33.69	0.6	35.45	31.29	<0.001
1995–2000	34.06	45.31	<0.001	40.03	52.50	<0.001
Maternal age at birth (mean, SD)	29.1 (5.2)	29.3 (5.4)	0.004	29.7 (5.4)	28.8 (5.4)	<0.001
Paternal age at birth (mean, SD)	32.2 (6.3)	32.2 (6.6)	0.6	32.5 (6.5)	31.8 (6.7)	<0.001
Birth country						
Sweden with a Swedish parent	78.93	88.33	<0.001	87.88	88.95	0.193
Sweden with foreign parents	14.96	9.66	<0.001	10.00	9.20	0.291
Outside Sweden	6.11	2.00	<0.001	2.12	1.85	0.449
Parental educational level, years in school						
⩽9	8.37	7.10	<0.001	6.07	8.51	<0.001
10–12	43.28	47.43	<0.001	45.08	50.62	<0.001
⩾13	48.35	45.47	<0.001	48.84	40.84	<0.001
Family income quintiles						
Lowest (1)	19.98	15.44	<0.001	15.56	15.28	0.76
Highest (2)	20.14	17.06	<0.001	18.53	15.05	<0.001
Mother had psychiatric history	34.54	52.39	<0.001	49.15	56.81	<0.001
Father had psychiatric history	22.27	31.74	<0.001	30.30	33.64	0.006
Compulsory school performance						
Qualification for upper secondary school						
At age 16	86.34	57.33	<0.001	61.96	52.39	<0.001
At age 20	88.91	66.26	<0.001	69.32	62.09	<0.001

ADHD: attention-deficit hyperactivity disorder; SD: standard deviation.

### Qualification for upper secondary education

Table 2 shows the RD for not qualifying for upper secondary education in relation to autism without ID at ages 16 and 20, overall and by sociodemographic characteristics including sex, birth country and income quintiles. Compared with non-autistic individuals, 29% more autistic individuals, 95% CI (28.0–30.0) were not qualified for upper secondary education at age 16. At age 20, the fully adjusted RD decreased to 22%, 95% CI (21.1–23.4). Females had a higher RD for failing to meet eligibility criteria for upper secondary education than males at ages 16 and 20. At age 16, 32% more females, 95% CI (29.9–34.3) were not eligible, compared to 27%, 95% CI (25.4–28.4) of males in the fully adjusted model. At age 20, the RDs decreased such that 26% more females, 95% CI (24.3–28.4) were not eligible as per 20% of males, 95% CI (18.7–21.4). Qualification for upper secondary education in autistic relative to non-autistic individuals also varied with immigration status, such that the RD was markedly lower among those born abroad. This variation was, however, largely explained by poor outcomes also in foreign-born individuals without autism (among whom 38 per 100 did not qualify at age 16) (Table 2). Autistic individuals with lower familiar income were more likely to fail to meet qualification criteria for upper secondary education relative to non-autistic individuals at both ages 16 and 20, than those with higher familiar income. At age 16, 29% (26.3–32.7) and 32% (29.4–34.7) more autistic individuals from the lowest and next-lowest familiar income quintiles failed to meet eligibility criteria, compared to 27% (24.1–29.4) and 24% (21.2–26.7) from the next-highest and highest quintiles. At age 20, the RDs decreased across income quintiles such that 24% (20.8–27.0) and 26% (23.0–28.0) more autistic individuals from the lowest and next-lowest familiar income quintiles failed to meet eligibility criteria, compared to 20% (17.6–22.5) and 17% (14.3–19.1) from the next-highest and highest quintiles.

**Table 2.** Non-qualification for upper secondary education at age 16 and 20 among students with autism spectrum disorders without intellectual disability (ID).

	At age 16				At age 20			
	Proportions %		RD (/per 100)		Proportions %		RD (/per 100 person)	
	No autism	Autism without ID	Model 1^a^	Model 2^b^	No autism	Autism without ID	Model 1	Model 2
Overall	13.7	42.7	29.0 (28.0, 30.0)	29.0 (28.0, 30.0)	11.1	33.7	22.6 (21.5, 23.8)	22.2 (21.1, 23.4)
Stratified by								
Sex								
Female	12.2	44.2	32.0 (30.0, 34.0)	32.1 (29.9, 34.3)	10.3	37.1	26.8 (24.7, 28.9)	26.4 (24.3, 28.4)
Male	15.1	41.9	26.8 (25.3, 28.3)	26.9 (25.4, 28.4)	11.9	32.1	20.3 (18.9, 21.7)	20.1 (18.7, 21.4)
Birth country								
Born in Sweden with Swedish parent(s)	10.4	41.8	31.4 (30.1, 32.7)	28.9 (27.6, 30.2)	8.4	32.8	24.4 (23.2, 25.7)	22.4 (21.2, 23.6)
Born in Sweden with parents born abroad	21.0	50.2	29.2 (25.2, 33.3)	30.1 (26.1, 34.2)	18.0	42.0	24.0 (20.0, 27.9)	24.7 (20.8, 28.7)
Born outside Sweden	38.4	45.5	7.2 (1.7, 16.0)	15.0 (6.1, 24.0)	29.0	35.8	6.8 (1.7, 15.3)	11.8 (3.7, 19.8)
Income quintiles								
0%–20% (1)	26.1	51.8	25.7 (22.5, 28.9)	29.5 (26.3, 32.7)	21.2	42.6	21.4 (18.2, 24.6)	23.9 (20.8, 27.0)
20%–40% (2)	17.4	50.0	32.7 (30.1, 35.3)	32.0 (29.4, 34.7)	14.6	40.6	26.0 (23.5, 28.6)	25.5 (23.0, 28.1)
40%–60% (3)	11.9	44.5	32.6 (30.1, 35.2)	31.0 (28.5, 33.5)	9.7	35.8	26.1 (23.6, 28.6)	24.8 (22.3, 27.2)
60%–80% (4)	8.2	36.0	27.8 (25.1, 30.5)	26.7 (24.1, 29.4)	6.4	27.3	20.9 (18.4, 23.4)	20.0 (17.6, 22.5)
80%–100% (5)	5.0	29.6	24.6 (21.9, 27.4)	23.9 (21.2, 26.7)	3.6	20.9	17.3 (14.8, 19.8)	16.7 (14.3, 19.1)

RD: rate difference.

95% Confidence intervals (lower limit, upper limit).

a Model 1: Adjusted for sex and birth year.

b Model 2: Adjusted for sex and birth year, birth country, immigration status, parental age and educational level at birth, family disposable income and parental psychiatric history.

### Comorbid ADHD

Table 3 shows the RD for not qualifying for upper secondary education among autistic students without ID with and without comorbid ADHD at ages 16 and 20. Comorbid ADHD increased the RD for not qualifying for upper secondary education among autistic students both at age 16 and 20, such that an additional 8%, 95% CI (5.1–10.2) and nearly 6%, 95% CI (3.4–8.2) failed to reach this outcome in fully adjusted models, respectively, as compared to autistic individuals without comorbid ADHD.

**Table 3.** Qualification for upper secondary education at ages 16 and 20 among students with autism without intellectual disability (Autism-ID), with and without comorbid ADHD.

	Age 16			Age 20		
	Proportion%	RD with 95% CI (per 100)		Proportion %	RD with 95% CI (per 100)	
		Model^a^	Model^b^		Model^a^	Model^b^
Qualification for upper secondary education						
Non-Autistic	86.3			88.9		
Autism-ID without ADHD	61.0	25.3 (23.8, 27.0)	25.8 (24.2, 27.4)	69.3	19.6 (18.1, 21.1)	19.7 (18.3, 21.2)
Autism-ID with ADHD	52.4	33.9 (32.0, 35.9)	33.0 (31.1, 34.9)	62.1	26.8 (25.0, 28.7)	25.8 (23.9, 27.6)
Comparison of Autism-ID without ADHD to Autism with ADHD						
		8.6 (6.1, 11.1)	7.6 (5.1, 10.2)		7.2 (4.8, 9.6)	5.8 (3.4, 8.2)

RD: rate difference; CI: confidence interval; ADHD: attention-deficit hyperactivity disorder.

95% CI (lower limit, upper limit).

a Model adjusted for sex and birth year.

b Model adjusted for sex, birth year, birth country, parental educational level and family disposable income at birth, parental psychiatric history and parental age.

### Core subject grades

Table 4 depicts proportions of individuals with non-passing grades in the three core subjects (Mathematics, Swedish and English) at ages 16 and 20, by diagnosis autism without ID and ADHD. Compared to the non-autistic, a larger proportion of the autistic group had non-passing grades in all core subjects at both ages 16 and 20; however, the proportions with non-passing grades in these subjects decreased by age 20. Total 39.1%; 95% CI (37.9–40.4) of 16-year-old autistic students without ID failed to achieve passing grades in Mathematics, compared to 12.2% (12.1–12.3) of those without autism. The proportion of students with not passing grades in Mathematics decreased at 20 to less than 29.1% (27.9–30.2) in autistic versus 9.3% (9.2–9.4) in non-autistic group. Similar results were observed in Swedish and English where more than one-third of all autistic students had non-passing grades compared to a tenth of those in non-autistic group at age 16. Numbers decreased at age 20 in both groups. Within the autistic group, students with comorbid ADHD presented higher proportions of non-passing grades in all core subjects at both 16 and 20.

**Table 4.** Proportions of individuals not passing core subjects (Mathematics, English and Swedish) at 16 and 20 years in relation to presence of autism without intellectual disability and ADHD, 95% CI.

	No-autism (%)	Autism-ID (%)	Autism-ID without ADHD (%)	Autism-ID with ADHD (%)
At 16 years				
Mathematics	12.2 (12.1, 12.3)	39.1 (37.9, 40.4)	35.9 (34.3, 37.5)	43.7 (41.7, 45.6)
Swedish	10.3 (10.2, 10.4)	35.3 (34.2, 36.6)	32.2 (30.7, 33.8)	39.7 (37.8, 41.5)
English	10.7 (10.6, 10.8)	34.3 (33.2, 35.5)	31.4 (29.9, 32.9)	38.4 (36.5, 40.3)
At 20 years				
Mathematics	9.3 (9.2, 9.4)	29.1 (27.9, 30.2)	26.7 (25.3, 28.2)	32.2 (30.4, 34.0)
Swedish	7.1 (7.1, 7.2)	23.9 (22.8, 24.9)	21.5 (20.2, 22.9)	27.0 (25.3, 28.7)
English	7.7 (7.6, 7.8)	22.7 (21.6, 23.7)	20.6 (19.3, 22.0)	25.5 (23.8, 27.2)

Autism-ID: autism without intellectual disability. ADHD: attention-deficit hyperactivity disorder; CI: confidence interval.

95% CI (lower limit, upper limit).

In Mathematics, 43.7% (41.7–45.6) of 16-year-old students with both diagnoses failed to reach passing grades compared to 35.9% (34.3–37.5) of those with autism without ID only.

## Discussion

In this large total-population study, we analysed eligibility for upper secondary education, defined by passing graduation grades in three core mainstream school subjects in students diagnosed with autism without ID. While many of these students met eligibility criteria for upper secondary education, they were considerably less likely to reach this outcome compared to their non-autistic peers (57.33% vs 86.34%). At age 16, which is the expected age for graduation in Sweden, 29% fewer students diagnosed with autism without ID were qualified for upper secondary education than non-autistic peers. This difference decreased somewhat at age 20. Comorbid ADHD, female sex and lower family income further increased the likelihood of students with autism without ID to not meet qualification criteria for upper secondary education, indicating the moderating role of theses covariates.

This study pertains to more recent birth cohorts, in whom diagnoses of autism without ID have become more common. Our results indicate that this group of youths, who commonly attend mainstream schools, does indeed have difficulties with qualifying for upper secondary education at expected age of graduation. These results confirm and expand previous studies with modest sample sizes, linking autism with educational underperformance (Keen et al., 2016; Levy & Perry, 2011). Previous research indicates that school performance in basic academic skills among pre-adolescents with autism without ID is similar to that of their typically developed peers (Goldstein et al., 1994). Yet, as adolescents they appear to underperform in areas related to language and mathematics (May et al., 2015). The latter is in line with the general pattern of underperformance seen among 16- and 20-year-old autistic individuals in this study. Individual IQ has been shown to partly predict academic outcomes in the general population (Gut et al., 2013). While our findings pertain to autism with IQ in the normal range and may indicate that factors beyond IQ underlie scholastic underperformance in those with autism, detailed data on IQ were not available to us to explore the role of IQ further. Previous research, however, showed discrepancies between individual achievement in academic skills and IQ in children with autism (Goldstein et al., 1994), and autism traits such as sensory deficits or social skills have been found to influence scholastic performance (Estes et al., 2011; Keen et al., 2016). Furthermore, we here presented objectively measured educational attainment of autistic students without ID in mainstream education where they are expected to perform alongside, as well as their non-autistic peers. Our results indicate that both IQ and factors beyond IQ should be studied in future research in order to gain detail knowledge on specific educational needs to help autistic students in attaining educational goals.

A noteworthy finding from our study is that autistic females were less successful in qualifying for upper secondary education than autistic males, such that additional 5% and 6% of females were not qualified in fully adjusted model at ages 16 and 20, respectively. This is in line with findings from a large, well-characterized study on autistic children that showed that females have lower cognitive and adaptive abilities, greater social communication impairments and externalizing problems compared to males (Frazier et al., 2014) indicating impairments that may contribute to scholastic achievement problems among females. Our findings are in sharp contrast to the patterns of higher school performance (Serbin et al., 1990) in girls in the general population and advantages in cognitive profiles in autistic girls observed in previous research (Bölte et al., 2011). Moreover, a study comparing parent and teacher reports of autistic symptoms in school-aged children found that teachers generally reported more symptoms in boys than girls and concluded that autistic girls have less difficulty adjusting to the school environment (Wong et al., 2015). While our study lacked detailed information on characteristics such as autism severity, cognition and psychopathological correlates that may contribute to sex differences in academic outcomes (Bölte et al., 2011; Frazier et al., 2014), we have previously shown that females were diagnosed at a later age than males in our cohort (Idring et al., 2012). Furthermore, there is evidence of a higher threshold for autism-diagnosis females (Nydén et al., 2000). We speculate that a delayed diagnosis, and hence possibly delayed educational support, as well as more severe symptoms may contribute to the sex discrepancy in school performance observed in our study.

In the general population, lower socioeconomic status and immigrant status are generally associated with poorer school outcomes (Bankston & Zhou, 2002; Sirin, 2005). This was also true for individuals with autism in line with reported patterns in educational outcomes later in life (Chiang et al., 2012, 2013). Yet, the difference between autistic and non-autistic group was less marked among foreign-born students as a high proportion also of non-autistic children failed to qualify for upper secondary education. Qualification for upper secondary school increased markedly, and in a similar manner, with family disposable income among autistic and non-autistic individuals in our data. Thus, higher family disposable income as an indicator of higher socioeconomic status does indeed buffer risks of school failure in both autistic and non-autistic students.

Over 40% of students with autism without ID in our study were found to have comorbid ADHD, a condition known to be associated with decreased scholastic attainments (Loe & Feldman, 2007). Indeed, comorbid ADHD further increased the likelihood of students with autism without ID to not meet criteria for upper secondary education, such that an additional 8% and 6% failed to meet these criteria at ages 16 and 20, respectively. To our knowledge, however, no previous study has examined the impact of ADHD on scholastic performance in autistic students. Identification and treatment of comorbid ADHD is important, considering that ADHD medication in non-autistic students has been found to improve some aspects of school performance (Baweja et al., 2015) and even increase likelihood of entering higher educational levels (Lu et al., 2017).

Qualifying for upper secondary school sets the stage for any student’s future educational achievements and labour market prospects (Chiang et al., 2012). Our findings that an increased proportion of students with autism without ID manages to qualify for upper secondary education at a later age (20 years) is therefore encouraging. This finding indicates that students with autism without ID have greater chance to qualify for upper secondary education if given an extended period of time to acquire this in mainstream compulsory schooling.

### Strengths and limitations

The strengths of this study include its total population design with prospective and rich data collected through register linkage on more recent birth cohorts. While ascertainment bias of autism without ID cannot be ruled out, such bias is likely to be small since we used multisource, validated case-finding approach (Idring et al., 2012). To our knowledge, this is the largest study to date on current school performance in mainstream settings in youth with autism without ID and the first large-scale study to also examine the role of comorbid ADHD. The study addresses educational outcomes among both adolescents and young adults with autism without ID, contributing to the knowledge on their eligibility for secondary education. This is also the first large study using actual administrative measures of school performance. Therefore, data generated in this study may be of broad relevance to individuals, families and society. The findings, however, need to be interpreted in the light of several limitations. Foremost, only data readily available in registers could be examined. We had no means to adjust results for individual IQs, which are suggested to predict educational outcomes in typically developed children (Deary et al., 2007). We were also unable to explore any mediating roles of factors such as specific autism traits (Culpin et al., 2018), or bullying which is commonly experienced by children with autism and may have influence on school performance. Similarly, data on the school type, including the pedagogical environment, together with teachers’ attitudes to inclusion and their skills to support and educate students with autism as well as type and level of health and education system support were unavailable to us. Maternal education level is commonly used in studies of child’s educational attainment and may have influence beyond broader covariate of *parents’ educational level at child’s birth* which was used in our study. Finally, although assessment of school performance is by law required to be fair and universal, inequalities in such assessments between students with and without autism may partly explain our findings (Avramidis & Norwich, 2002). Hence, equality in evaluations of school performance in those with neuropsychiatric conditions needs attention in future studies. In addition, further analyses of risk and resilience factors, including the importance of the school environment (e.g. pupil–teacher ratios and availability of teachers with training in special needs education) should be addressed in future studies.

## Conclusion

The results of this contemporary total population study indicate that individuals with autism without ID, especially those with comorbid ADHD and girls, are significantly less likely to reach qualification for upper secondary education than their non-autistic peers at the expected age of graduation. Although indicating some improvement compared to previous studies on educational outcomes, examining older autistic cohorts, our findings underline the need for further identification of these students and supportive interventions for them during mainstream compulsory school. They may also be viewed as challenging the inclusion education policy adopted by majority of western countries (OECD Education Policy Analysis (Deluca, 2003)), aimed at pursuing equity for these students.

On a more hopeful note, however, our findings that more than half of all students with autism without ID in mainstream schools do qualify for upper secondary education at the expected age of graduation, and that this proportion increases at a later age (20 years), are encouraging and emphasize the importance of considering extended primary schooling in youth with autism without ID.
